# Effects of pre-notification, invitation length, questionnaire length and reminder on participation rate: a quasi-randomised controlled trial

**DOI:** 10.1186/s12874-017-0467-5

**Published:** 2018-01-05

**Authors:** Marie Koitsalu, Martin Eklund, Jan Adolfsson, Henrik Grönberg, Yvonne Brandberg

**Affiliations:** 1 Department of Oncology-Pathology, Karolinska Institutet. Karolinska University Hospital, Z1:00, 171 76 Stockholm, Sweden; 2 Department of Medical Epidemiology and Biostatistics, Karolinska Institutet, Boxes 281, 171 77 Stockholm, Sweden; 3 Department of Clinical Science Intervention and Technology, Karolinska Institutet, 171 77 Stockholm, Sweden

**Keywords:** Participation rate, Response rate, Pre-notification, Invitation letter, Questionnaire length, Reminder

## Abstract

**Background:**

Improving participation rates in epidemiologic studies using questionnaires and biological sampling is important for the generalizability of the outcome. The aim of this study was to examine the effects of pre-notification, invitation length, questionnaire length, and reminder on participation rate and to investigate whether some factors contributed to participants doing both the questionnaire and blood sampling as oppose to only one part.

**Methods:**

Our study was embedded within the pilot testing of a large population-based study about prostate cancer screening. Our study sample consisted of 28.134 men between 50 and 69 years of age and living in the region of Stockholm (Sweden) invited to respond to a web-based questionnaire and to provide blood for prostate cancer testing. The men were randomly allocated according to birth of date to receive either: (a) a pre-notification postcard or not; (b) a shorter or a longer invitation letter; (c) a shorter or a longer web-based questionnaire, and (d) a reminder or not. The effects of the survey design factors were tested using chi-square.

**Results:**

The use of a pre-notification (*p* < 0.0001), a longer questionnaire (*p* = 0.004) and the use of a reminder (*p* = 0.02) were associated with an increase in overall participation, i.e. responding to the questionnaire or providing blood for PCT or performing both components.

**Conclusions:**

The results of this pilot study justified the use of a pre-notification and a reminder in the following large population based study since the benefits of increased participation traded off against the greater costs incurred. Furthermore, we were able to use the longer version of the questionnaire, which allowed us to collect more information without risking a lower response rate.

**Electronic supplementary material:**

The online version of this article (10.1186/s12874-017-0467-5) contains supplementary material, which is available to authorized users.

## Background

Web-based surveys offer many advantages for improving data quality compared to written questionnaires [1]. Skipping irrelevant sections which are conditional on the responses to previous questions, minimising invalid responses, and requiring answers to missed questions are examples of how web-based surveys may increase the rate of complete surveys [2]. Not only are they improving data quality, but also, more cost-effective than postal or telephone surveys [3]. This is especially attractive for large population studies since material and staff costs tend to be proportional to the number of respondents. In addition, with Internet access being now widespread, barriers to electronic data gathering are diminishing.

At the same time, participation in epidemiological studies has decreased over the last few decades [2, 4]. Refusal rates are increasing, whereas making contact with potential respondents is becoming more strenuous [5]. For surveys directed to specialised populations such as professional groups or students, sending an e-mail with a direct hyperlink is usually possible. However, the lack of such email lists for the general population entails for an initial contact to be made through the use of conventional mail and not e-mail.

A large body of research has sought to identify various factors possibly affecting participation rates [5–7]. Factors affecting internet response rates show general similarities to those found for other survey modes [6]. Response rates for web-based questionnaires have been reported to be lower than for paper-based questionnaires [8]. Nonetheless, given the rapid changes within electronics and internet-literacy, and as suggested by Hohwü et al. [9], the platform used to collect data should reflect the development of electronic devices. Hence, web-based questionnaires could replace paper-based questionnaires with minor effects on response rates [9]. Thus, there is a need to examine participation rates in large population studies using a log-on web-based questionnaire coupled with conventional mail as mode of initial contact.

Between 2012 and 2014, a large population-based study of prostate cancer testing was carried out in Stockholm, Sweden [10]. Initial contact was made by conventional mail, inviting participants to log-on to a web-based survey and to provide blood sample for diagnostic measurements of prostate cancer risk. Because the study aimed to invite over 100.000 men, concerns were raised over the optimal invitation procedures.. Those procedures included use of a pre-notification card, length of the invitation letter, length of the questionnaire and use of a reminder. The aim of the study was to compare participation rates between the different modes of invitation and recruitment approaches.

## Methods

### Study context

The study was embedded in the pilot study for the Stockholm 3 trial (STHLM3), a large population-based diagnostic study of men aged 50–69 years investigating prostate cancer testing (PCT) [10]. The pilot study was conducted in order to test the feasibility of the invitation and survey design, as well as the procedures for data collection. Participation consisted of two components: completion of a web-based questionnaire and provision of a blood sample for prostate cancer testing (PCT). PCT required a visit to a hospital or a health centre for blood sampling. The participants could either decline to partake, or participate in one or both components. The PCT risk estimates were based on the blood samples only. The answers to the web-based questionnaire were collected to amount information about the participants in terms of physical activity, diet, knowledge about prostate cancer in the population, attitudes toward PCT, and health related quality of life.

### Study sample and procedures

Invitations were sent out between September 2012 and November 2012 during six consecutive weeks to a total of 28.134 men without a previous prostate cancer diagnosis, between 50 and 69 years of age, living in the region of Stockholm. They were randomly selected by date of birth from the Swedish Population Register, kept by the Swedish Tax Agency. The invitations were sent on a weekly basis to all men who met the criteria and who had their birthday that same week, thus rendering this sampling quasi-randomised.

The men in the study sample were allocated to receive: (a) a pre-notification postcard or not; (b) a shorter or a longer invitation letter; (c) a shorter or a longer web-based questionnaire, and (d) a reminder or not. The allocations were combined into six study arms. Each arm represents one dispatch week and the distribution is presented in Table 1.

**Table 1:** Distribution of men randomized to each of the six modes of allocation

Study Arm	Pre-notification	Invitation	Questionnaire	Reminder	N
1.	Yes	Short	Short	Yes	3575
2.	Yes	Long	Short	Yes	3601
3.	Yes	Short	Short	No	6130
4.	Yes	Short	Long	No	4835
5.	No	Short	Long	No	4956
6.	No	Short	Short	No	5037
Total					28,134

### Dependent variables

Data on provision of blood samples and on questionnaire completion were collected from the STHLM3 database. The outcomes measured were the proportion of completed web-based questionnaires, the proportion of men who provided blood samples, and the proportion of participants who completed both. The dependent variables were assessed by the end of April 2013.

### Independent variables

#### Pre-notification

The pre-notifications were sent out as postcards one week before the mailed invitation letters. The postcard described the forthcoming invitation to participate in the STHLM3 trial, and, thereby, getting a free PCT, as well as more information about STHLM3.

#### Postal invitation letter

Both the short and the long invitation letters consisted of one sheet of paper. The front page contained information about the study, as well as the individual credentials to login to the web-survey, and stated that the web-based questionnaire would take approximately 20 min to fill out. The long version (406 words in Swedish) was approximately twice as long as the short version (218 words in Swedish). The back of the sheet contained a checklist explaining what to bring when giving the blood sample and how to respond to the web-survey. The checklist was made out of 274 words and was included in both the short and the long version of the invitation letter. Along with the letters, an extensive brochure with more in-depth information about the STHLM3 study and its procedures were included in both versions of the letters. Consequently, both invitation packages contained all in all the same information. The differences consisted in the format of the letter and the amount being mentioned in the front page of the letter. Both letters had the login information of the front page. An English translation of the letters can be found in the supplementary files (see Additional file 1).

#### Web-based questionnaire

The questionnaire was designed for the study and largely similar to the one used later on in STHLM3 [10]. The short web-based questionnaire consisted of 500 items; whereas, the long web-based questionnaire consisted of 1000 items. The short web-based questionnaire took an average of 18 min to fill out, whereas, the long web-based questionnaire took, on average, 47 min to complete. The layout and the issues tapped by the long and the short questionnaires were similar. The differences laid in how many follow-up or sub-questions there were.

#### Reminder

Those who were assigned to receive a reminder were sent a reminder two weeks after receiving the invitation letter, regardless of whether they had already participated or not. Apart from stating that it was a reminder, the letter used the same wording as the short invitation letter.

### Data analysis

Descriptive statistics were performed to present the study sample. The participation rates between the different arms were compared by Chi-square tests. All tests were two-sided and significance level was set to 0.05.

## Results

Out of the 28.134 men invited to participate, 9.543 men (34%) participated to one or two components. Of those participants, 7.302 men (77%) participated in both components, i.e., responded to the web-based questionnaire and provided blood for PCT; 1.744 men (18%) only provided blood for PCT; and 497 men (5%) only responded to the web-based questionnaire.

**Fig. 1:**
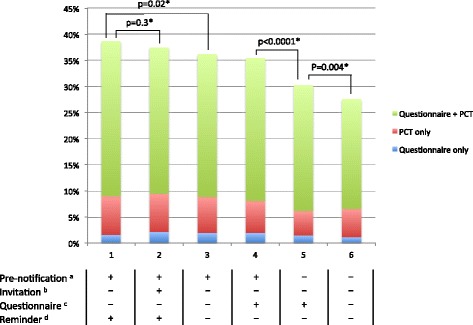
Participation rates for each dispatch arms. * *P*-values of participation to one or two components are shown. ^a^ + = presence of pre-notification; **−** = absence of pre-notification. ^b^ + = long invitation letter; **−** = short invitation letter, ^c^ + = long questionnaire; **−** = short questionnaire. ^d^ + = presence of reminder; **−** = absence of reminder

Use of a pre-notification (*p* < 0.0001), a longer questionnaire (*p* = 0.004) and a reminder (*p* = 0.02) was associated with an increase in overall participation, i.e. participation to one or two components. The length of the invitation letter was not associated with participation (Fig. 1).

Additionally, out of the 7.302 men that participated to both components, pre-notification (*p* = 0.0007) and a longer questionnaire (*p* = 0.0003) were associated with an increase in participation.

Finally, among the 1.744 men who only responded to the questionnaire, none of the four factors were associated with participation. Pre-notification was the only factor that increased participation (*p* = 0.002) among the 497 men who only provided blood for PCT.

## Discussion

In the present study, inviting almost 30,000 men, associations on participation rates between four invitation and survey design factors (pre-notification, invitation letter length, questionnaire length, and reminder) were investigated within the STHLM3 setting. These four factors have been shown to play a substantial role in influencing response rates for surveys in previous studies [6, 7]. Use of a pre-notification, a reminder and a longer questionnaire were associated with increase in overall participation, whereas the length of the invitation did not appear to have any impact.

Sending a pre-notification turned out to be associated with increased participation rates. This finding is in concordance with previous research, stating that pre-notifications increase the odds of response [11]. However, we do not know whether that increase is due to the content of the postcard, the format of the postcard, or the pre-notification itself. The sentence “Join the study STHLM3 and get a free prostate cancer test”, which was included in the pre-notification postcard, could be seen as an incentive, and could have contributed to the positive association.

The length of the invitation letter was not associated with participation rates. This could be due to the fact that the overall information provided in the invitation packages was similar for both the short and the long invitation letter. The difference laid in the format of the front page, and the amount of information delivered on that front page. Hence, it seems that the participants receiving the shorter invitation letter were given enough information.

Previous population-based studies suggest an inverse association between questionnaire length and response rate [11–13]. Conversely, in our study, the longer questionnaire was associated with higher participation rates. A possible explanation is that the men who spent longer time responding to the questionnaires were more involved and the items might have led to a greater interest in participation in PCT. Another possible explanation as to why questionnaire length did not show a negative association with response rate in our study could be in the statement of the length of the web-based questionnaire in our invitation letter. Galesic et al. [14] manipulated the stated length and showed that a longer stated length reduces the response rate. In our study, however, 20 min was the stated length for both questionnaires. Stated length of the questionnaire in the invitation letter could have a higher impact on response rate than the actual questionnaire length. Thus, it is likely that once a man had started to respond to the questionnaire, he completed it despite the amount of time required.

A web-based questionnaire was used, and the Swedish population can be considered as a highly Internet-literate population with almost full access to the Internet. Additionally, according to de Bernardo et al. 2013 [15], web survey as a tool of data collection is as effective as paper surveys in populations above 50 years of age. Therefore, it is unlikely that the participation rates were affected by a lack of computer knowledge or by a lack of access to Internet.

The participation rates were higher for PCT than for the web survey, even though the PCT required attendance to a medical centre, whereas the web survey could be completed at home or at work. A possible explanation for the higher participation rates for PCT is that the men perceived a personal benefit of participating in PCT. By participating they received information about their individual risk for prostate cancer whereas responding to the questionnaire did not result in any benefit for the individual man.

The strengths of the present study include the large sample, the randomized design and the use of the STHLM3 database, where reliable data were available. In addition, the questionnaires used were based on international studies on PCT.

The results from this study on factors of importance for participating in a PCT study were used for the STHLM3 main trial and seemed to substantially having increased the participation rates. The rates increased from 34% observed in the present pilot study to 42% achieved in the large population based STHLM3 study [10]. The results also highlight the importance to test, when possible, the study design of an upcoming trial, especially if it will be delivered to a large population sample.

## Conclusion

The results of this pilot study suggest that web-based questionnaires coupled with pre-notification may be an alternative to costly traditional paper questionnaires, and that there is room for improvement in response rates.

The results of this pilot study justified the use of a pre-notification and a reminder in the following large population based study since the benefits of increased participation traded off against the greater costs incurred. Furthermore, we were able to use the longer version of the questionnaire, which allowed us to collect more information without risking a lower response rate. No negative effects on participation rates were observed by the use of the longer invitation letter or the longer questionnaire. The results of this study were used in the STHLM3 study, resulting in an improvement of the participation rate.

## Additional file

Additional file 1: English translation of the invitation letters as well as the checklist. A. Long version of the invitation letter. B. Short version of the invitation letter. (PDF 184 kb)

## Abbreviations

PCT: Prostate cancer testing
PSA: Prostate specific antigen
