# Supplementary material for: Effects of pre-notification, invitation length, questionnaire length and reminder on participation rate: a quasi-randomised controlled trial
Source: BMC Med Res Methodol. 2018 Jan 5;18:3. doi: 10.1186/s12874-017-0467-5 (PMC5756335; doi:10.1186/s12874-017-0467-5)
Supplement: Additional file 1: — English translation of the invitation letters as well as the checklist. A. Long version of the invitation letter. B. Short version of the invitation letter. (PDF 184 kb) [file 12874_2017_467_MOESM1_ESM.pdf]

1. A. Longer version of the invitation letter

Do you want to join STHLM3 and test new diagnostics for prostate cancer?
Karolinska Institutet studies, in STHLM3, better diagnostics for prostate cancer.

To take part in STHLM3 mean that:

- You read through the attached brochure and decide whether you want to participate
- You provide a blood sample of 12 ml
- You respond to a survey about yourself and your lifestyle. The survey takes approximately 20 minutes to respond
- You get a free prostate cancer test followed by an answer within 3 weeks

You join STHLM3 by within two weeks go to one of the laboratories* that are part of STHLM3 to provide a blood sample. The sampling takes approximately 5 minutes and you do not need to make an appointment. It is important that you bring the consent referral that you have received in this letter as well as an ID with a swedish personal identification number. When you have provided a blood sample and signed the referral you are part of STHLM3. A laboratory that is located near your residence is for example: [Name of nearest laboratory], [Address of nearest laboratory]

To respond to the survey you go to www.sthlm3.se and fill in your username [studyID] and your password [password]. You can as well answer to the survey before you provide your blood sample.
Everyone who participate in STHLM3 will within 3 weeks from the blood sampling get an answer of their own risk for prostate cancer. The answer will be sent by mail to the address where you are registered. Depending on the test result you will get one of the three following recommendations:

- You have a low risk to get prostate cancer. We recommend a new test in 10 years.
- You have a normal risk to get prostate cancer. We recommend a new test in 2 years.
- You have an elevated risk for prostate cancer. We recommend that you meet an urologist for further investigation and biopsy of the prostate.

If you happen to have an elevated risk of prostate cancer, STHLM3 will make sure you get a referral for further examination with an urologist and biopsy of the prostate. We will send you contact details to the urologists who participate in STHLM3. You will also get more information about what biopsy means. However, we ask you to notice that you have to book the appointment with the urologist that you choose to contact.

If you have any questions, you are welcome to contact STHLM3 by phone 08 – 524 86 797 or by e-mail sthlm3@ki.se.

Sincerely,

Henrik Grönberg, Research leader
Professor, Karolinska Institutet

> Please check our checklist for participation and our FAQ at the back of this invitation

* Se attached list: “Participating laboratories” to find the place that is the best for you

B. Shorter version of the invitation letter

Do you want to join STHLM3 and test new diagnostics for prostate cancer?
STHLM3 is the worlds largest scientific study about prostate cancer and its goal is to develop better diagnostics for prostate cancer. Karolinska Institutet and Stockholms County Council is inviting all men in Stockholm between 50-69 years to join STHLM3.

To take part in STHLM3 mean that:

- You read through the attached brochure and decide whether you want to participate
- You provide a blood sample of 12 ml
- You respond to a survey about yourself and your lifestyle. The survey takes approximately 20 minutes to respond
- You get a free prostate cancer test followed by an answer within 3 weeks

Everyone who participate in STHLM3 will within 3 weeks from the blood sampling get an answer of their own risk for prostate cancer. The answer will be sent by mail to the adress where you are registered. Depending on the test result you will get one of the three following recommendations: low risk, normal risk or elevated risk for prostate cancer.
If you happen to have an elevated risk of prostate cancer, STHLM3 will make sure you get a referral for further examination with an urologist. For further information se the attached brochure.

If you have any questions, you are welcome to contact STHLM3 by phone 08 – 524 86 797 or by e-mail sthlm3@ki.se.

Sincerely,
Henrik Grönberg, Research leader
Professor, Karolinska Institutet

Your username to the study is [studyID] and your password [password].

A laboratory that is located near your residence is for example: [Name of nearest laboratory], [Adresse of nearest laboratoy]

If you choose to join: look at the checklist for participation as well as the FAQ on the back of this invitation.

Checklist for participating in STHLM3

If you want to be part of the STHLM3 study, follow this checklist:

1. Providing blood sample
   You join STHLM3 by within two weeks go to one of the laboratories that are part of STHLM3 to do a blood test.
   The blood test takes approximately 5 minutes and you do not need to book an appointment. You can go to any of the listed laboratories on the attached list. On the first page of this letter we have proposed one that is located near your residence.
   When visiting the laboratory it is important that you bring with you: the consent referral that is attached to this letter and an ID with a Swedish personal identification number.
2. Responding to the survey
   Responding to the survey takes approximately 20 minutes and you do that on the web. Go in to www.sthlm3.se and click on the link "To the survey". Write your username and your password which you can find on the first page of this letter.
   You can respond to the survey before you do your blood test.

Frequently asked question and answer about STHLM3

- Do I have to join? No, this is an offer. You do not need to join if you do not want to. If you do not wish to be part of this you do not need to do anything.
- What should I do with the attached consent referral? You should bring the attached consent referral with you to the laboratory when you are doing your blood test.
- I would like to join STHLM3 but I have not had time to go within two weeks. Can I still join? Yes, but we would prefer that you go within two weeks.

More questions and answer can be found in the attached brochure and on www.sthlm3.se.
